# Copper biosorption by *Bacillus pumilus* OQ931870 and *Bacillus subtilis* OQ931871 isolated from Wadi Nakheil, Red Sea, Egypt

**DOI:** 10.1186/s12934-023-02166-3

**Published:** 2023-08-12

**Authors:** Amal William Danial, Fatma Mohamed Dardir

**Affiliations:** 1 Botany and Microbiology Department, Faculty of Science, Assiut University, Assiut, Egypt; 2 Geology Department, Faculty of Science, Assiut University, Assiut, Egypt

**Keywords:** Biosorption, Cu^2+^, *Bacillus*, Black shale

## Abstract

**Background:**

Despite being necessary, copper is a toxic heavy metal that, at high concentrations, harms the life system. The parameters that affect the bioreduction and biosorption of copper are highly copper-resistant bacteria.

**Results:**

In this work, the ability of the bacterial biomass, isolated from black shale, Wadi Nakheil, Red Sea, Egypt, for Cu^2+^ attachment, was investigated. Two Cu^2+^ resistance *Bacillus* species were isolated; *Bacillus pumilus* OQ931870 and *Bacillus subtilis* OQ931871. The most tolerant bacterial isolate to Cu^2+^ was *B. pumilus*. Different factors on Cu^2+^ biosorption were analyzed to estimate the maximum conditions for Cu biosorption. The q_max_ for Cu^2+^ by *B. pumilus* and *B. subtilis* determined from the Langmuir adsorption isotherm was 11.876 and 19.88 mg. g^−1^, respectively. According to r^2^, the biosorption equilibrium isotherms close-fitting with Langmuir and Freundlich model isotherm. Temkin isotherm fitted better to the equilibrium data of *B. pumilus* and *B. subtilis* adsorption. Additionally, the Dubinin-Radushkevich (D-R) isotherm suggested that adsorption mechanism of Cu^2+^ is predominately physisorption.

**Conclusion:**

Therefore, the present work indicated that the biomass of two bacterial strains is an effective adsorbent for Cu^2+^ removal from aqueous solutions.

## Introduction

Due to their high toxicity, nonbiodegradability, and bioaccumulation in living cells, heavy metals found in wastewater are of great interest [1–6]. Heavy metals are dispersed into the environment by a variety of companies, including those that create batteries, textiles, dyes, tanneries, plastics, glass and smelting. Insecticide use, sludge use, and municipal garbage releases all have an adverse influence on the water and soil [7–9]. High copper concentrations have been linked to numerous physiological issues, health issues, and even mortality [10]. Ions of copper are regarded as hazardous substances [11]. Additionally, consuming too much copper damages the liver and gallbladder and affects the human metabolism [12]. As a result, copper is one of the main heavy metals that requires direct removal using various approaches such as nano technological applications, i.e. development of nano-sized materials, tubes and composites as adsorbents have engrossed rapidly [13, 14]. For both environmental and business reasons [15, 16], it is crucial to extract and recover heavy metals from wastewater [17]. Previous studies focused on reviewing the most advanced wastewater treatment techniques, including adsorption, membrane filtration, cementation and electrodialysis [18].

Heavy metals can be difficult to get rid of from aquatic systems. The microbial cell is intriguing in this regard and has received a lot of attention as a cheap and effective adsorbent [19–21].

In a controlled environment, it is appropriate to use chemical or physical means to obtain free microbial cells or enzymes, as this promotes adsorbent activity and makes microbial cell recycling easier. High copper concentration in drinking water was linked to *Variovorax* sp. biofilms in the system of copper plumbing, and the ability of bacteria to cause microbial induced corrosion was observed [22–25]. Numerous studies showed that, compared to suspension cells, immobilized microbes improve hazardous chemical tolerance and breakdown [26–28]. An intriguing possibility is the use of bacterial leftovers as an adsorbent for heavy metals ions [12]. However, since wastewater's copper (II) concentration is less than 10 mg. L^−1^, Cu ^2+^ removal is complicated and results in an unproductive wastewater concentration when discharged.

The Langmuir and Freundlich models of the adsorption isotherm are two that are frequently used. The Langmuir model was initially proposed to describe how gas molecules adsorb onto uniform solid surface (crystalline materials) that exhibit one specific sort of adsorption site as in Langmuir [29]. Several studies deal with copper removal from aqueous medium [30–33]**.**

To bridge the knowledge gap in the field of study, this work aimed to elucidate the role of native bacteria (*Bacillus pumilus* and *B. subtilis*) in bioremediation of copper which isolated from Wadi Nakheil, Red Sea, Egypt. Exploration of copper resistant bacteria from the Red Sea has not been widely carried out. Since the number of bacteria strains resistant to heavy metals, is quite limited. It was expected that the new copper resistant bacterial isolates as copper bioremediation agent candidates will be obtained to handle the problem of environmental pollution. Factors affecting biosorption and mechanisms of biosorption were also studied. Biosorption isotherms and kinetics parameters were determined from biosorption measurements. Bacterial species are considered efficient and cost effective for treating wastewater containing heavy metals. This work also, provides a comprehensive overview of mechanistic studies of heavy metal removal from aqueous solutions using various modeling methods and would bridge the gap between laboratory experiments and field data, thus providing a reliable environmental assessment of wastewater treatment applications.

## Results and discussion

**Fig. 1:**
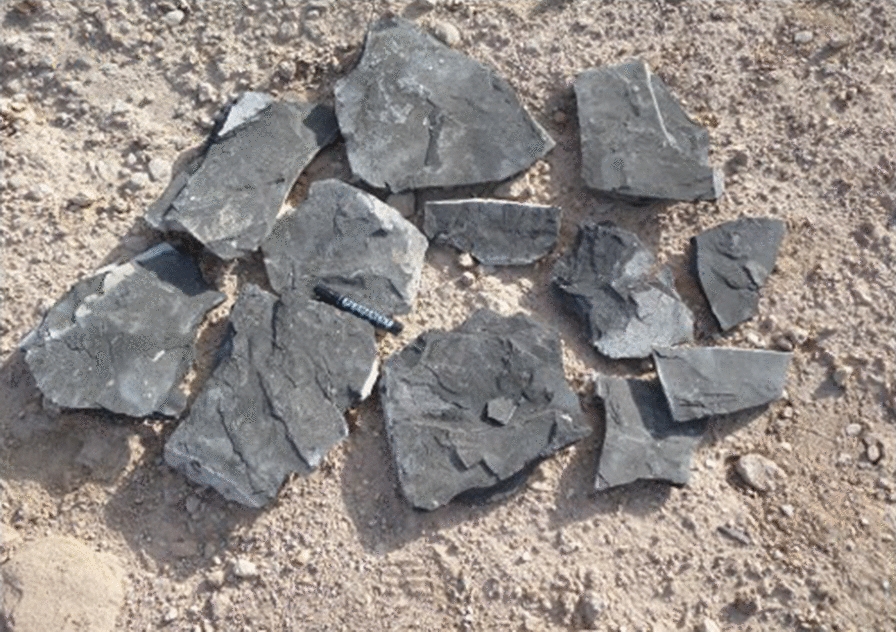
Black shale waste dumped nearby Nakheil

Black shale samples were collected from Wady Nakheil, Red Sea, Egypt (Fig. 1). The sample is rich with total organic compounds (TOC); it contains 23% TOC. El Kammar [34] reported that Dakhla Formation consists of organic-rich calcareous shale to argillaceous limestone that can be considered as a good to excellent source rock potential and the total organic carbon (TOC) content ranged from 2.04 to 12.08%. Parviainen and Loukola-Ruskeeniemi [35] concluded that black shales are sedimentary rocks containing > 0.5% of organic carbon and they are host to some mineral such as Cu, Ni, Zn, Mn and P. Copper (Cu^2+^) is perhaps the most prominent metal produced from black-shale-associated Kupferschiefer ore [36]. Samples of black shales extracted from “gypsum” mines, in Ipubi member of the Santana Formation include mineral and high organic matter, the high content of organic matter evidence the hydrocarbon potential of these shales [37].

### X-ray diffraction

**Fig. 2:**
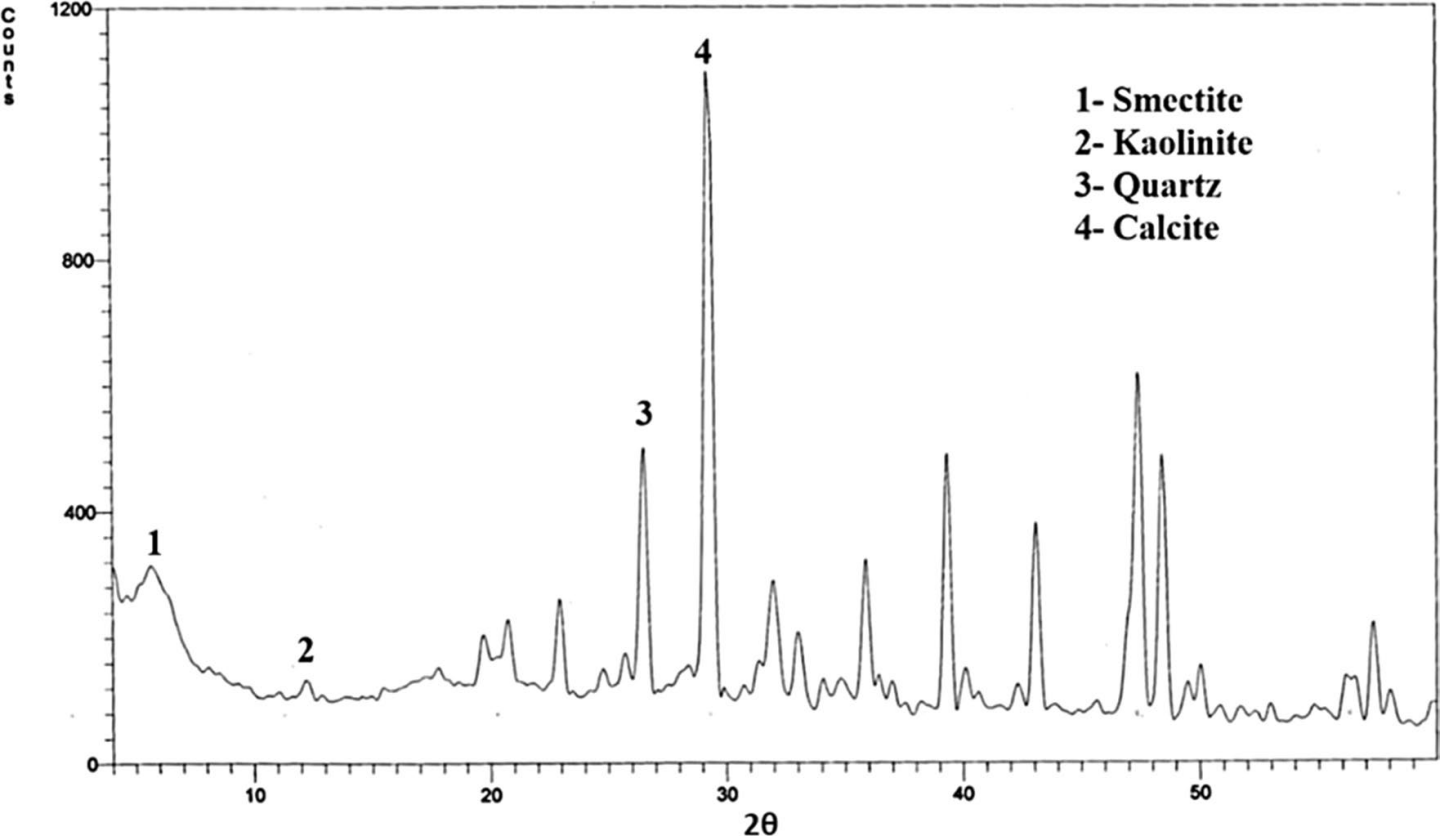
XRD diffractogram of waste black shale, Dakhla Formation, W. Nakheil, Quseir area

X-ray diffraction of the black shale sample was scanned, and the sample consisted of calcite, quartz and traces of smectite and kaolinite (Fig. 2). Similar results were observed by Zhigang et al. [38] who found that black shale of southeastern Ordos Basin was deposited of quartz, feldspar, carbonate and clay minerals. El Aouidi et al. [39] reported that Black Shale from the Ama Fatma Coastal Site in the Southwest of Morocco were composed essentially of calcite (up to 70%). The predominance of carbonates in the sedimentary environment revealed that the deposition occurred in open marine settings.

In marine black shale, the carbonate deposit resulted from microbially mediated anaerobic oxidation of methane in the shallow subseafloor at a hydrocarbon seep [40]. The black shales from the uppermost member of the Duwi Formation in the Qusseir area are composed of montmorillonite, kaolinite, calcite, gypsum, quartz and pyrite [41].

### Thermal analysis

**Fig. 3:**
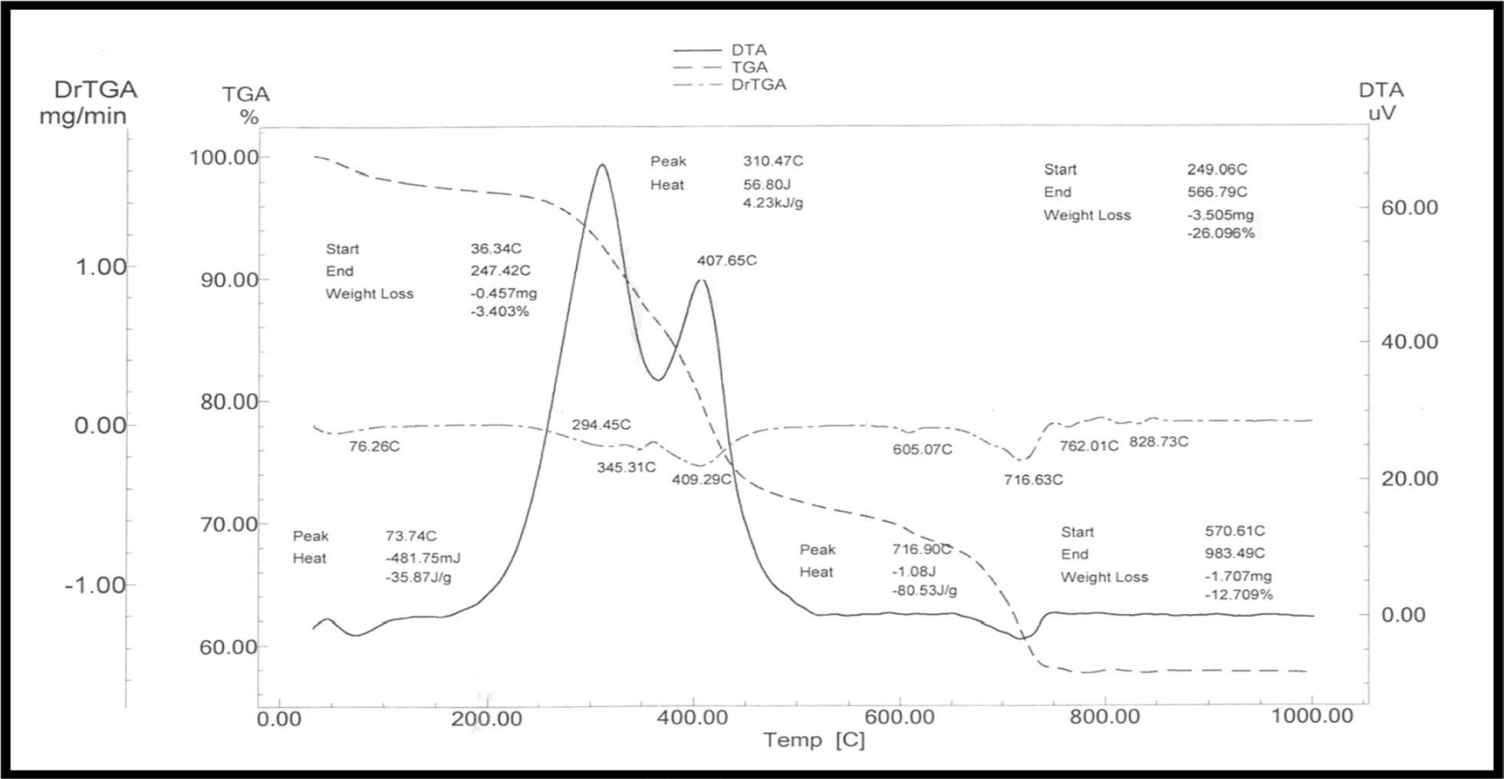
TG and DTA curves of black shale waste, W. Nakheil, Quseir area

Thermal study of the thermogravimetry (TG) and differential thermal analysis (DTA) curves revealed that the volatile hydrocarbon from black shale may be responsible for weight loss of about 42%. The breakdown of carbohydrates and silica may be the cause of weight loss that is seen at temperatures exceeding − 650 °C. The samples predicted energy evolution was 1012 kcal/Kg. The potential of the energy is therefore rather good to use this sample as an energy source, as can be inferred from the finding (Fig. 3). Thermal treatment of Estonian graptolite– argillite samples made emission of water, carbon dioxide, sulphur dioxide, nitrogen oxides, and different hydrocarbon fragments [42]. Non-isothermal kinetics and thermal analysis of *Gerçüş* tar sand sample, thermogravimetry (TG/DTG) curves of tar sand samples at different particle sizes demonstrated three stages of weight loss [43].

### Fourier—transform infrared (FT-IR) spectroscopy

**Fig. 4:**
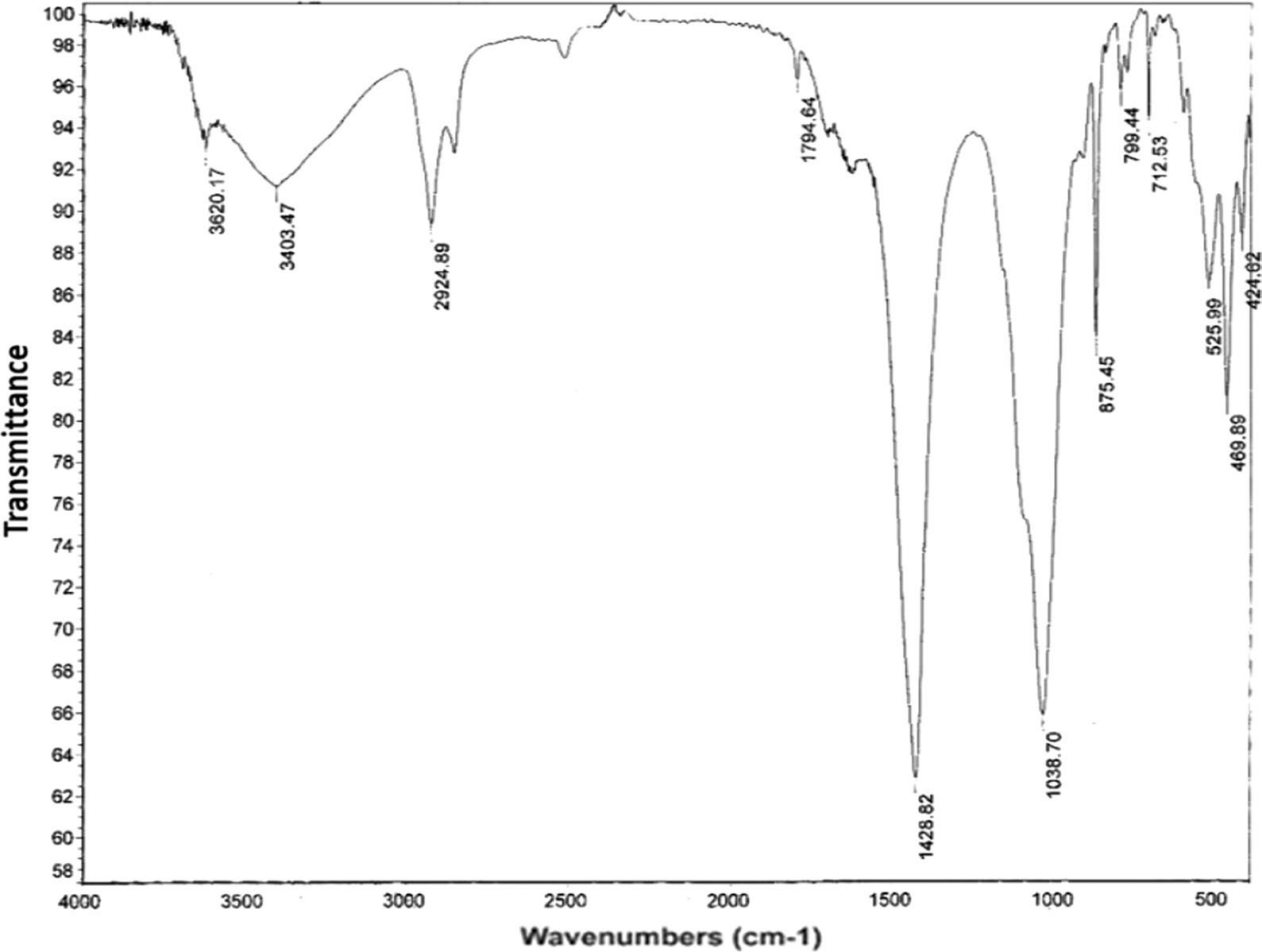
FT-IR spectrum of black shale, W. Nakheil, Quseir area

The well-defined distinct bands seen in FT-IR examinations (Fig. 4) at 3403.4 cm^−1^, a distinctive band that defines the OH group may be found. The C-H stretching group is associated with the sharp band that appears at 2924.8 cm^−1^ [44].

The C-H and/or C-OH groups are associated to the strongest peak in the spectra, which were found at 1428.8 and 1038.7 cm^−1^. There is a connection between the calcite mineral and the strong peak at 875.4 cm^−1^.

These spectral features reveal that the bacteria were indeed adsorbed onto black shale. Similar results were obtained by Sethurajan et al. [45], suggesting enhancement in its bioleaching efficiency for the extraction of copper.

### Surface area characteristics

The surface area of the waste black shale sample was found to be 4.15 m^2^. g^−1^. The average pore diameter observed was 98.234 Å.

Previous studied concluded that clay’s surface area affects the values of Arrhenius constant, while it is the catalytic properties of clay, which lower the activation energies of all the reactions involved in the combustion process [46]. Black shale organic material internal surface area decreases dramatically during OM dissolution from ∼15 m^2^. g^−1^ to ∼5 m^2^. g^−1^ [47].

### Isolation, identification of copper-resistant bacteria

**Fig. 5:**
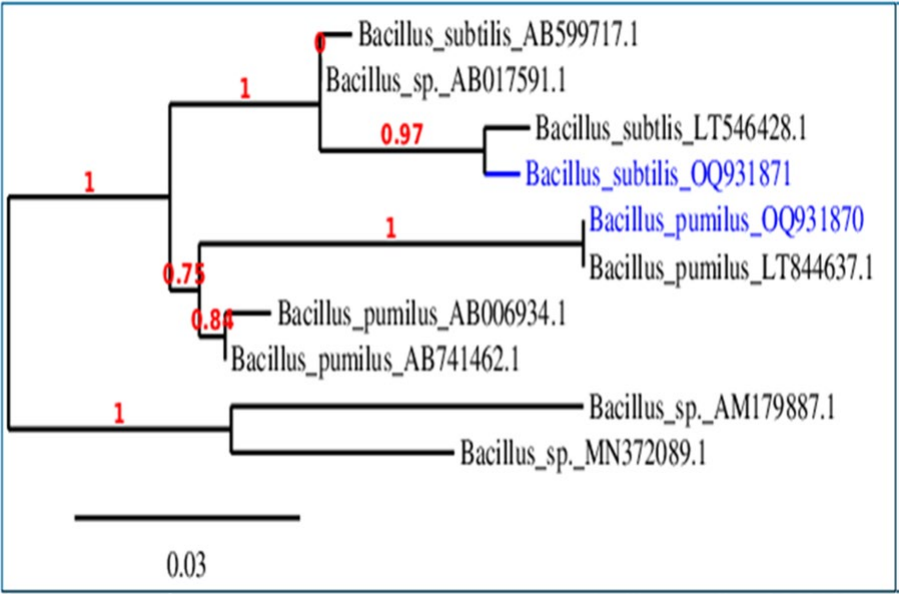
Phylogenetic tree on the basis of patterns and genetic relationship of *Bacillus pumilus* (B1) and *Bacillus subtilis* (B2)

Bacterial isolates were identified based on morphology (Table 1) and genetical characterization. According to the 16S rRNA, and comparison in NCBI GenBank databases, the result showed that strain B1 had 98% nucleotide base homology to *Bacillus pumilus* and B2 had 100% homology of *Bacillus subtilis* (Fig. 5).

**Table 1:** Morphological and biochemical characteristics of metal resistant soil bacterial isolates

Test	*Bacillus pumilus*	*Bacillus subtilis*
Gram stain	+	+
Shape	Rod	Rod
Spore formation	+	+
Motility	+	+
Nitrate reduction	–	+
Urease	–	–
Catalase	+	+
Citrate	–	+
Oxidase	+	–
Indole	–	–
MR	+	–
VP	+	+
Gelatine hydrolysis	–	+
H_2_S	–	–
Oxidation and fermentation of:		
Glucose	+	+
Sucrose	+	+
Mannitol	+	+
Fructose	+	+

Bacteria of the genus *Bacillus* are Gram-positive rods. The cell wall of bacteria is the first construct in the cell which contacts copper ions during biosorption mechanisms, such as ions exchange, complexation, coordination, chelation, precipitation [48, 49]. Daughney and Fein [50] who worked with *B. subtilis* and *B. licheniformis* concluded that the different in sorption sites concentrations due to the change in cell wall structure, because both bacteria can produce spores, which have strong wall which is different from non-spore forming bacteria in structure [51]. Moreover, the impact of ions which present in higher salinities solution cause a reduction in the metal’s removal, due to the competition for biosorption sites on the cell.

Wong et al. [52] revealed the metal uptake ability by describing the isolation of *Micrococcus* sp, from sludge of local wastewater treatment facility which survive several cycles (at least five) of copper biosorption and desorption.

Different strategies of microbial metal (copper) resistance or tolerance well known are bioaccumulation or sequestration. Others include exclusion, compartmentalization, and complexation by binding proteins [53].

### Effect of temperature, pH, and copper concentration on bioremediation and growth

**Fig. 6:**
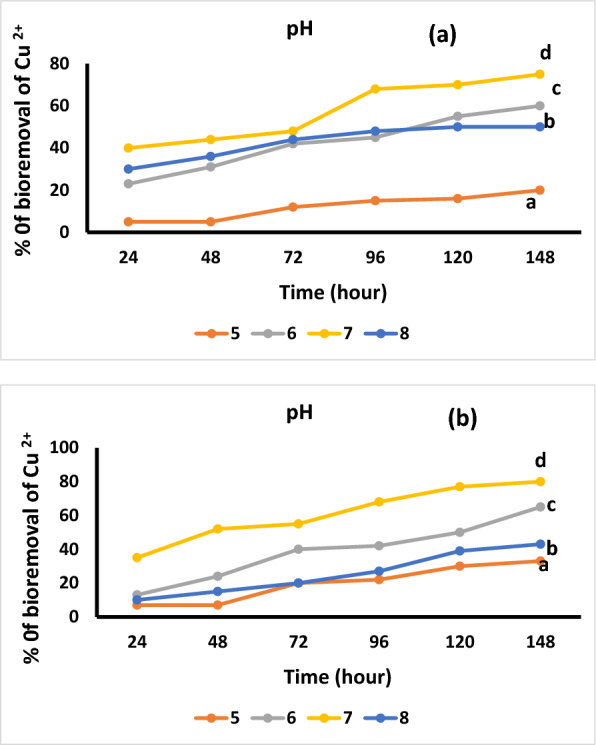
Effect of pH on Cu ^2+^ adsorption by *Bacillus pumilus* (**a**) and *Bacillus subtilis* (**b**). The ANOVA test was carried out by using SPSS 21 comparisons among means (n = 3), different letters show significance among the different treatments at p = 0.05 level based on Duncan's multiple range test

The effect of pH on the adsorption of Cu ^2+^ by *Bacillus* isolates was estimated at concentration of 20 mg. L^−1^, 30 min, and 30 °C as shown in Fig. 6a, b. The figure demonstrated that, increasing the pH of solution from 5.0 to 8.0, the percentage of adsorption increased. The data showed the higher pH for adsorption of Cu^2+^ of *B. pumilus* and *B. subtilis* were 7.0. However, for pH more than 8.0, Cu^2+^ precipitated. Precipitation of Cu^+2^ at higher levels of pH disturbs the process of adsorption, so the metal will not be available for biosorption. The lower efficacy to remove metal ions at low pH is acceptable by the competitive biosorption between H^+^ and metal. At higher pH, there are more acidic and functional groups, and they attract Cu ^2+^ by biomass of bacteria [54]. Earlier findings showed that optimum pH to biosorption of Cu^2+^ by *Micrococcus* and *Pseudomonas* 5.0 [55, 56]. Vijayaraghavan and Yun [2] indicated that the pH-depend on the copper ions in the biosorption methods, attributed to the negative charge of cell wall and physicochemical effects, such as metal hydrolysis. According to previous experiments, bacterial cell walls contain carboxyl groups, amides and amines which protonated or deprotonated depending on the liquid media pH [51, 57].

**Fig. 7:**
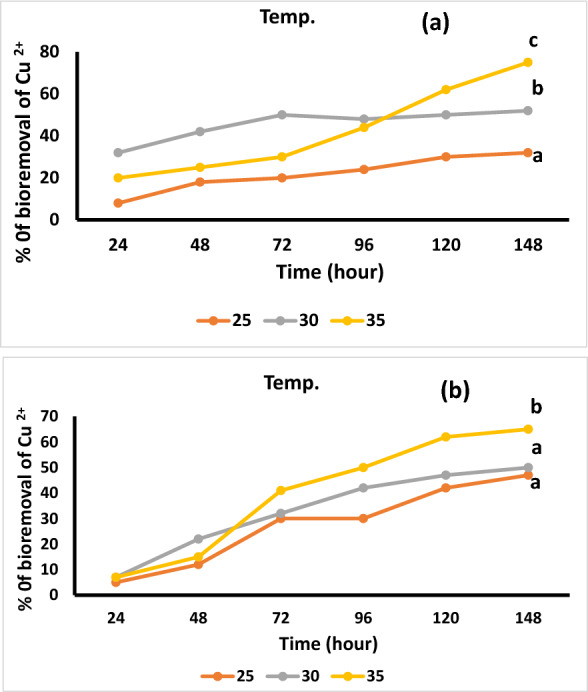
Effect of temperature on Cu ^2+^ adsorption by *Bacillus pumilus* (**a**) and *Bacillus subtilis* (**b**). The ANOVA test was carried out by using SPSS 21 comparisons among means (n = 3), different letters show significance among the different treatments at p = 0.05 level based on Duncan's multiple range test

Effect of temperature on Cu^2+^ by two *Bacillus* isolates was significantly increased by increase the temperature from 25 to 35 °C. the result showed that highest temperature for Cu^2+^ adsorption was at 35 °C for *B. pumilus* and *B. subtilis* (Fig. 7a, b).

Interface between biomass and the metal ions affects temperature, by influencing the strength of the metal–sorbent complex, and the ionization of the cell-wall moieties. Biosorption solution temperature could be helpful in mechanisms dealing with energy in metal-binding process [58]. In contrast, is energy-independent mechanisms. Previous studies showed that biosorption of copper ions by *Bacillus* Species are less affected by temperature because the responsible processes to remove metals are largely physicochemical in nature [56].

**Fig. 8:**
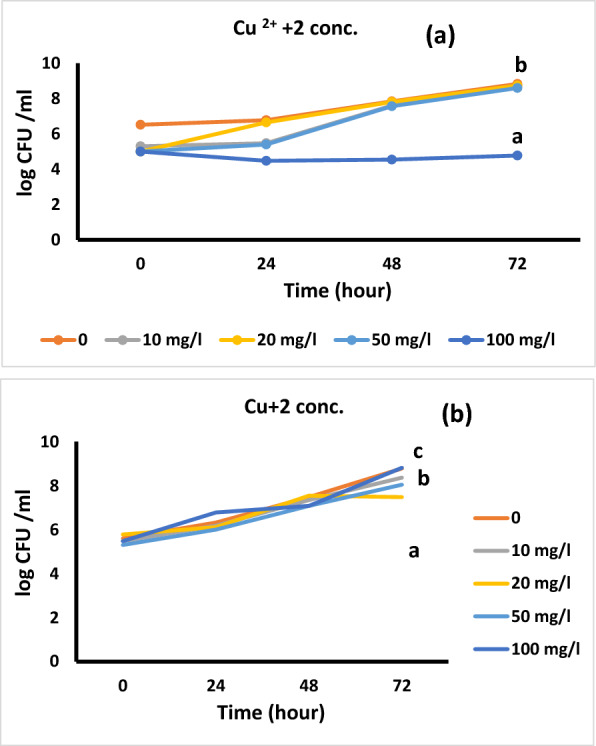
Effect of copper concentrations on CFU of *Bacillus pumilus* (**a**) and *Bacillus subtilis* (**b**). The ANOVA test was carried out by using SPSS 21 comparisons among means (n = 3), different letters show significance among the different treatments at p = 0.05 level based on Duncan's multiple range test

The concentration of copper effect on colony forming unit (CFU) of *Bacillus* isolates is shown in Fig. 8a, b, the most optimum concentration was 20 and 50 mg. L^−1^ Cu^2+^ for the two isolates at 72 h old cells. These results were highly significance.

Studying the adsorption time of copper using dead bacterial cells has high effect in removal of metal, because it depends on the adsorbent nature. It is believed that using mesophilic microorganisms, metal-ions adsorption by dead cells, which is metabolism-independent passive binding of cell walls, extends equilibrium at 5:15 min [56].

Higher uptake of metal at low dry mass amount due to increasing of metal/biosorbent ratio, which decreases with an increase the concentration of biosorbent [56]. S-layer surface and paracrystalline are important bacterial traits that manipulated for metal binding and envelop existing in several bacteria. This layer regarded as interface between cell and environment, is produced by glycoprotein or protein monomers that can self-assemble in two-dimensional structures. In Gram positives, the attachment of peptidoglycan to cell wall, makes up a porous protein compound of the same morphology and size.

At 20 mg. L^−1^copper concentration, the removal percentages were increased to 45 and 30% for *B. pumilus* and *B. subtilis*; respectively which is significantly difference between the two isolates (Table 2). The maximum biosorption capacity of Cu ^2+^ ions on *Bacillus sp*. were determined to be 16.25 ± 1.64 mg. g^−1^ [59]. Copper uptake by *Micrococcus* sp. was examined by Wong et al. [52] who described that 36.5 mg of copper (II) per gram dry weight is taken at pH 5.0, while 15 mg is taken at pH 6.0. Nakajima et al. [60] using alkaline and solvent treated *Micrococcus luteus* reported a similar amount of 33.5 mg copper per gram dried cells. Also, the biomass of inactivated cells can use up significant amounts of copper [61], and the cell wall of bacteria is also active in cation binding [62]. Several bacteria, due to their additional compartment, need to deal with both cytoplasmic and periplasmic copper [63]**.**

**Table 2:** percentage of bioremoval of copper by *Bacillus pumilus* and *B. subtilis* at 30 °C

Cu^+2^ concentration(mg/l)	*Bacillus pumilus*	*Bacillus subtilis*
0	0	0
10	33b	25ab
20	45c	30b
50	22a	18a
100	0	0
150	0	0

The ANOVA test was carried out by using SPSS 21 comparisons among means (n = 3), different letters show significance among the different treatments at p = 0.05 level based on Duncan's multiple range test

Copper adsorption by *Enterococcus faecalis* and *Pseudomonas aeruginosa* gave efficiency rates up to 90% [64]**,** while *E. coli* BL21 RIL strain had a copper accumulation efficiency 4.79 mg. L^−1^ of culture normalized at an optical density of 1.00, which was 1250 times more efficient than the control strain [65]. Also, copper quantities accumulated by *Enterobacter cloacae* IrSuk1, *Enterobacter cloacae* IrSuk4a, and *Serratia nematodiphila* IrSuk13 are of 0.96, 0.85 and 1.89 mg. gram^−1^ dry weight of cells; respectively [66].

### Determination of minimum inhibitory concentrations (MIC) for copper

The MIC of Cu^2+^ for *B. pumilus* OQ931870 (B1) and *Bacillus subtilis* OQ931871 (B2) isolates were 300 and 330 μg.ml^−1^, respectively, (Table 3). *B. pumilus* was a highly tolerant isolated bacterium. The existence and abundance of resistant metal microorganisms in polluted locations are studied in previous works [67, 68]. It seems that prolonged term uses up of sewage sludge have utilized selection pressure on populations of soil by microorganisms and recognized their metal resistance [69].

**Table 3:** Minimum inhibitory concentration (MIC) of metals for resistant bacteria

Metal	MIC (mg/l)	
	*Bacillus pumilus* (B1)	*Bacillus subtilis* (B2)
Cu ^2+^	300	330

### Biosorption experiments and kinetics of sorption

A reasonable fit was confirmed using the corresponding coefficient of determination (R2) values, which were utilised to estimate various parameters of the tested kinetic models.

**Fig. 9:**
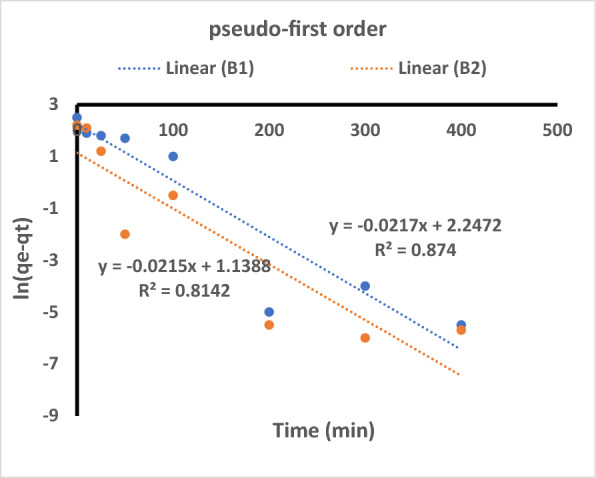
The linear form of the pseudofirst order equation for Cu^2+^ by *Bacillus pumilus* and *Bacillus subtilis*

With low R2 values (R2 > 0.9, Table 4, Fig. 9), both PFO models demonstrated a satisfactory lack of fit to the experimental data for *B. pumilus* and *B. subtilis* biosorption.

**Table 4:** Different parameters for the first-order, second order, Elovich and intraparticle diffusion models for the biosorption of copper by *Bacillus pumilus* and *Bacillus subtilis*

Models	Parameters	*B. pumilus*	*B. subtilis*
Experimental data			
First order model	q_e_^exp^ (mg g^−1^)	9.23	3.02
	K_1_ (min^−1^)	0.0217	0.0215
	q_e_^cal^ (mg g^−1^)	9.461	3.123
	R^2^	0.874	0.814
Second order model	q_e_^exp^ (mg g^−1^)	4.12	1.01
	K_2_ (g mg^−1^ min^−1^)	0.009	1.237
	q_e_^cal^ (mg g^−1^)	4.366	0.918
	R^2^	0.946	0.999
Elovich model	β	0.382	0.479
	α	0.243	0.147
	R^2^	0.977	0.964
Intraparticle diffusion	K_i_ (mg g^−1^ min^−1/2^)	0.23	0.287
	R^2^	0.914	0.91

**Fig. 10:**
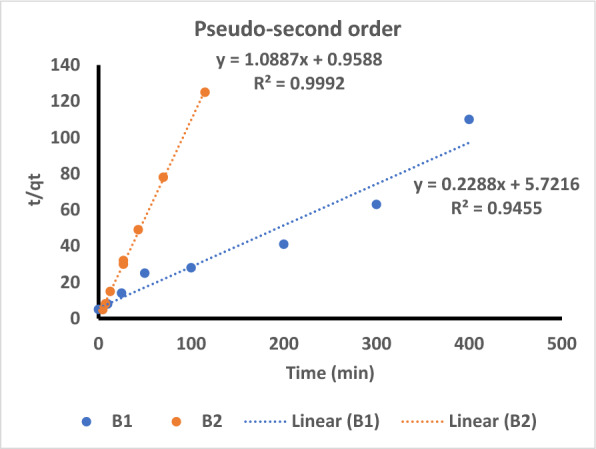
The linear form of the pseudosecond order equation for Cu^2+^ by *Bacillus pumilus* and *Bacillus subtilis*

The pseudo second order (PSO) model, however, showed a superior fit. Additionally, the q_e_ value and the value predicted by the PSO model correspond well (Fig. 10). The PSO kinetic model suggests a rate-controlling mechanism through the formation of chemical bonds between the adsorbent and adsorbent and adsorbate molecules, whereas the pseudo first order (PFO) kinetic model assumes that the adsorption phenomenon is controlled by the mass transfer process due to differences in the adsorbate concentration between the surface of the adsorbent and the solution [70–72].

Because of this, the adsorption of B1 and B2 was more likely to be chemical. The calculated K2 values for B1 were lower than those for Be, confirming that B1 adsorbs faster than B2 (Table 4). This difference in adsorption rates may be due to different adsorbent properties.

The Elovich model, in contrast, presupposes that the active adsorbent sites are heterogeneous and are distinguished by a range of sorption energies.

**Fig. 11:**
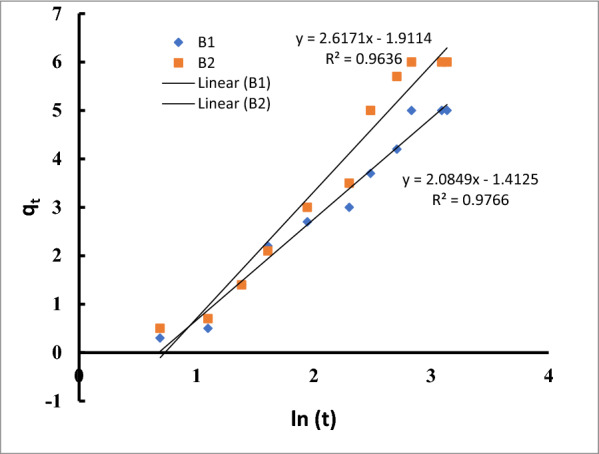
The linear form of the Elovich equation for Cu^2+^ by *Bacillus pumilus* and *Bacillus subtilis*

The findings showed that for both B1 and B2, the Elovich model had excellent R2 values (R2 >  0.96, Table 4, Fig. 11).

The Elovich equation was therefore appropriate, sufficient, and best specified for the adsorption of drugs on the surface of bacteria.

**Fig. 12:**
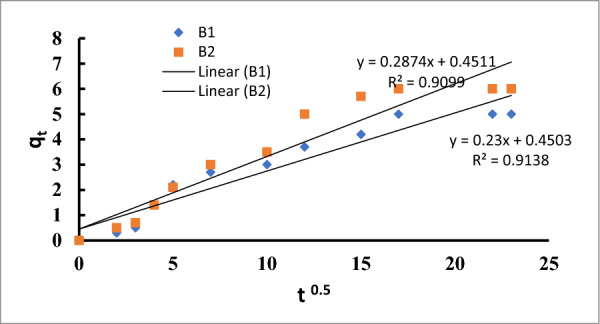
The linear form of the Intraparticle diffusion equation for Cu ^2+^ by *Bacillus pumilus* and *Bacillus subtilis*

The chemisorption rate and surface coverage were determined by measurements, respectively. In the case of B1, the reading was lower than in case of B2, and vice versa. The adsorption mechanism and rate-limiting step are typically described by the intraparticle diffusion equation. For the two studied strains, the intra-particle diffusion model fit their data well (R2 = 0.91) (Table 4, Fig. 12). Indicating that the intraparticle diffusion mechanism was significant in the mass transfer from the aqueous solution to the surface of the bacterium, the plot of qt vs. t 0.5 for B1 and B2 was linear.

The boundary layer effect was, however, verified by the plot's departure from the origin, which revealed that intra-particle diffusion was not the rate-controlling mechanism [70].

Khan et al. [73] concluded that the efficient removal of Cu^2+^ by *B. altitudinis* MT422188 was 73 mg. L^−1^ and 82 mg. L^−1^ of Cu^2+^ at 4 and 8 day intervals. *Bacillus* sp. 5O5Y11 was found to have high tolerance to a group of heavy metals (Fe, Cu, Pb, Ag, Zn) [74]. *Bacillus amyloliquefaciens* BSL16 showed significantly higher bioremediation potential by accumulating high Copper [75].

*Microbacterium paraoxydans* strain VSVM IIT(BHU) presented a maximum heavy metal ion removal efficiency of 91.62% Cr (VI), 89.29% Pb (II), and 83.29% Cd (II) at 50 mg/L [76].

### Adsorption isotherms

Due to electrostatic attraction and the abundance of active sites on the material, metal adsorption kinetics accelerates during the initial phase of interaction between the adsorbent and adsorbates [71, 72, 77]. The surface of adsorption of adsorbents properties of adsorbents and the model of adsorption isotherms used to examine how adsorbents and adsorbate adsorb similarly [78, 79].

To study metal adsorption, Langmuir and Freundlich isotherm models were evaluated. *Bacillus* isolates data on the isotherm biosorption of Cu ^2+^ using Langmuir and Freundlich isotherms [73–75, 80].

**Fig. 13:**
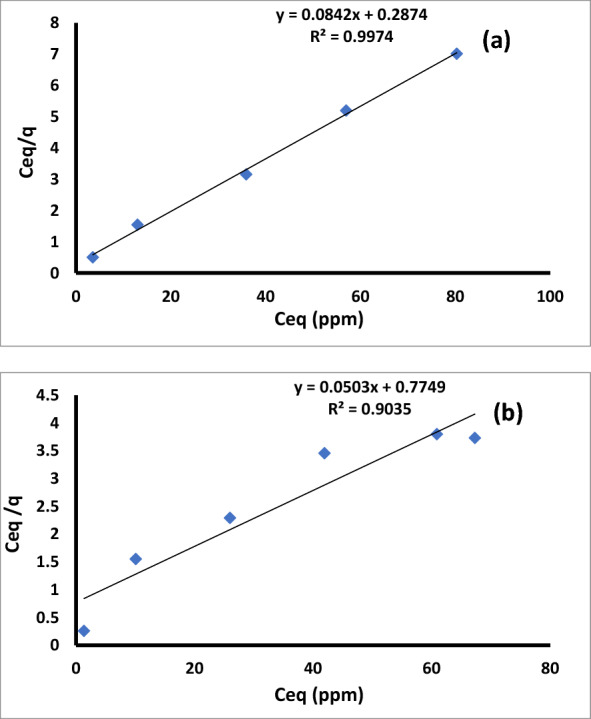
The linear form of Langmuir adsorption isotherm of Cu^2+^ by *Bacillus pumilus* (**a**) and *Bacillus subtilis* (**b**)

*Bacillus* isolates were used to measure the Cu^2+^ biosorption at starting values of 0–100 mg. L^−1^ for 72 h and pH range of 5.0–8.0 (Table 5; Fig. 13a, b).

**Table 5:** Calculated parameter for different isothermal for the adsorption of copper by *Bacillus pumilus* and *Bacillus subtilis*

Models	Parameters	*B. pumilus*	*B. subtilis*
Langmuir	q_m_ (mg. g^−1^)	11.876	19.88
	b (L. mg^−1^)	0.2929	0.0649
	R^2^	0.9974	0.9035
Freundlich	1/n	0.164	0.257
	K_F_ (mg. g^−1^ (L. mg^−1^) ^1/n^)	5.66	4.569
	R^2^	0.9348	0.9235
Temkin	A_T_ (L. mg^−1^)	0.0031	0.0027
	b_T_ (J. mol^−1^)	43.9	50.27
	R^2^	0.943	0.93
Dubinin and Radushkevich	β × 10^−8^ (mol^2^. J^−2^)	2	2
	E (KJ. mol^−1^)	5	5
	R^2^	0.987	0.964

The graph demonstrates the relationship between the concentration of remained metal ions in solution and the quantity of metal ions that two *Bacillus* isolates adsorbed. Using the Langmuir model, the data were fitted (Fig. 13a, b). The Langmuir parameter values were presented in Table 5. *B. pumilus* and *B. subtilis* have q_max_ values of 11.876 and 19.88 mg. g^−1^ for Cu^2+^ biosorption, respectively.

According to the findings, *B. pumilus* was able to biosorb more copper (II) than *B. subtilis*. The variation of chemistry ions could be used to explain the preference of bacteria for the biosorption of Cu ^2+^ [81].

**Fig. 14:**
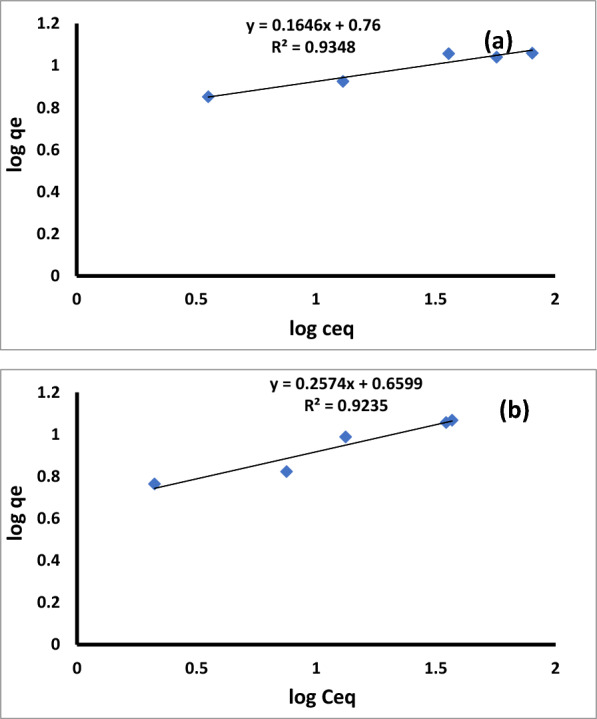
The linear form of the Freundlich adsorption isotherm equation for Cu.^2+^ by *Bacillus pumilus* (**a**) and *Bacillus subtilis* (**b**)

For Cu ^2+^, the biomass b values for B1 and B2 are 0.2929 and 0.0649 l. mg^−1^, respectively. The Freundlish isotherm's linear form was mentioned elsewhere in a previous study [79, 82]. Figure 14a, b illustrate the linear version of the Freundlich equation for the biosorption of Cu ^2+^ by B1 and B2 (Fig. 14a and b).

The sorption capacities of the biomass surfaces improved with rising initial copper concentrations, according to Freundlich isotherms. Table 5 displays the values of the Freundlich parameters.

Table 5 also, showed the magnitude of K_f_ and n, with B1 adsorbing more Cu^2+^ than B2. For Cu^2+^ for B1, the greatest K_f_ and n values were 5.66 and 6.075334, respectively. Table 4 implies that n is greater than unity, showing that B1 and B2 preferentially adsorb copper ions. The strong correlations demonstrated that the models accurately represent the biosorption equilibrium of Cu^2+^ by the B1 in the concentration range under investigation.

**Fig. 15:**
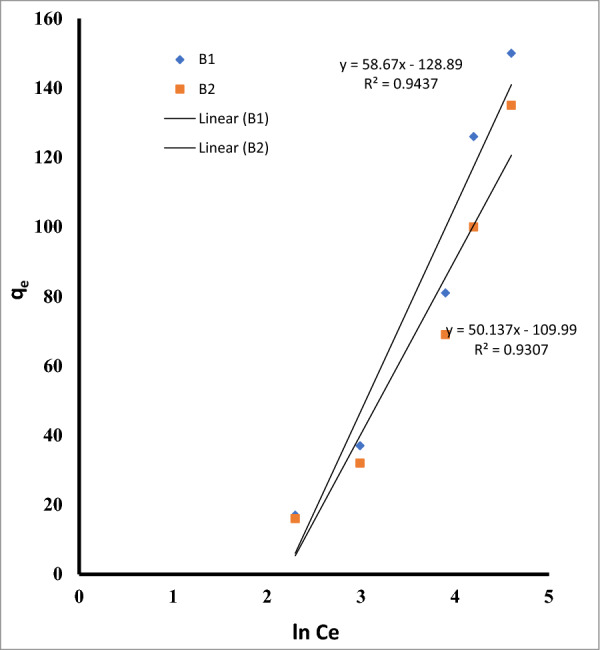
The linear form of the Temkin adsorption isotherm equation for Cu ^2+^ by *Bacillus pumilus* and *Bacillus subtilis*

**Fig. 16:**
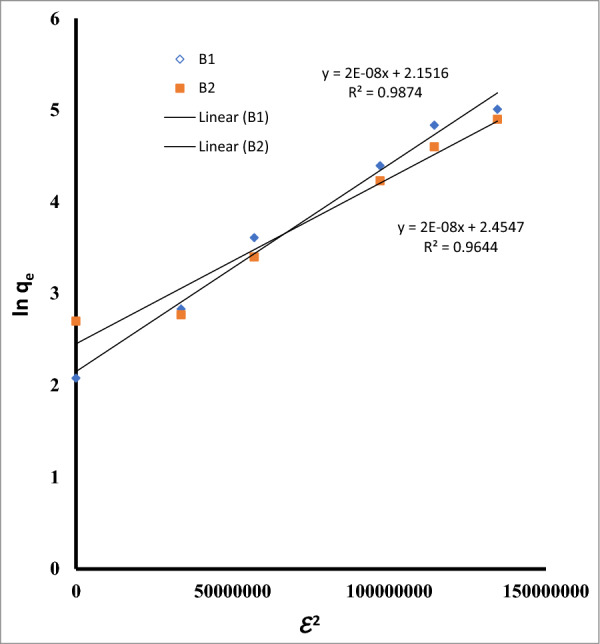
The linear form of the D-R adsorption isotherm equation for Cu ^2+^ by *Bacillus pumilus* and* Bacillus subtilis*

The Temkin isotherm further supported the existence of heterogeneous active sites with various binding energies by acceptable match to the experimental data. Additionally, the D-R isotherm suggests that b1 and B2 fit well (Table 5, Fig. 15). Based on the D-R data, it can be determined if the adsorption process is chemisorption (E > 16 kJ mol^−1^) or physisorption (E 8 kJ mol^−1^). The mean free energy of adsorption (E, KJ mol^−1^) is connected to the energy carried from the adsorbate molecules to the surface. Because both B1 and B2's predicted E values in the current investigation were less than 8 (Table 5, Fig. 16), it seemed likely that physisorption was the primary adsorption process [70].

## Materials and methods

Black shale samples were collected and crushed (< 250 mesh) and exposed to X-ray diffraction (XRD), Differential thermal analysis (DTA), Fourier—transform infrared spectroscopy (FT-IR) and surface area analyses at Assiut University.

### X-ray diffraction

To determine the mineralogical composition of the studied bulk waste black shale sample, it was scanned between 4 and 60° 2θ using X-ray diffractometer generated at 40 kV and 30 mÅ, Model PW 1710 control unit Philips Anode Material Cu, optics: Automatic divergence slit with Cu Kα radiation λ = 1.5405°A over a wide range of Bragg angles (20° ≤ 2θ ≤ 90°).

### Thermal analysis

The TGA and TG Analysis were made using simultaneous TG–DTA apparatus thermal analyzer (Shimadzu DTG-60H). The experiments were performed between ambient and between 200 and 500 °C. The temperature program had a heating at 310 °C for the first stage and 407 °C for the second step.

### Fourier—transform infrared spectroscopy (FT-IR)

FT—IR examinations with a Nicolar spectrophotometer (model 6700). The instrument used for this analysis was a Nicollet 6700 FT-IR equipped with data station (Assiut University, Egypt, Thermo Fisher Scientific, 168 Third Avenue, Waltham, MA 02451, USA).

### Surface area properties

The surface area of the waste black shale sample was measured by using liquid Nitrogen adsorption/desorption isotherms nitrogen at − 196 °C by the Quantachrom Instrument Corporation, USA (Model Nova 3200). The surface area of the studied sample was found to be 4.15 m^2^/g. The average pore diameter observed was 98.234 Å.

### Isolation of copper-resistant bacteria

Samples were collected from black shale, Wadi Nakheil, Red Sea. Samples inoculated in medium (Luria–Bertani (LB) supplemented with 100 mg. L^−1^ Cu^+2^). Then incubated at 30 °C for 7 days, then inoculated into fresh medium containing 100 mg. L^−1^ copper. The cells were collected by centrifugation for 10 min (6000 rpm) and washed twice with 0.85% NaCl. The cell pellet was freeze dried, and its dry weight was measured. The dried cells were digested with concentrated nitric acid (70%). The obtained cultures were examined for their capacity to remove copper by atomic absorption spectrophotometer (AAS) (Analytik Jena, Germany). The higher copper removal ability culture was isolated. Culture was purified on LA medium supplemented with 100 mg. L^−1^ Cu, and pure colonies be seen were streaked on fresh LA medium.

### Bacterial identification

Morphological and biochemical tests for Gram stain, gelatin hydrolysis, nitrate reduction, oxidation–fermentation of glucose, oxidase test, catalase, indole and urease production were performed as described by Barrow and Feltham [83], motility, and fermentation of glucose, mannitol, sucrose. The isolated bacteria were classified using methods mentioned by Hussein et al. [84].

### Genetical identification

The primer used was “F (5′-AGA GTT TGATCC TGG CTCAG-3′)” with a GC clamp and “R (5′-GGT TAC CTT GTTACGACT T-3′)” at the annealing temperature of 65 °C were used for the PCR amplification of the variable region of 16S ribosomal DNA (rDNA) from the purified genomic DNA. The PCR product is made at a Korean company by ABI 3730xl DNA sequencer. Sequence analysis was conducted based on the online databases using BLAST by MEGA 3.1 software for phylogenetic analysis. The neighbor- joining method was used to build the phylogenetic tree.

### Nucleotide sequence accession numbers

The nucleotide sequence of the bacterial isolate B1 (*Bacillus pumilus*) and B2 (*Bacillus subtilis*) were deposited in the database of GenBank nucleotide sequence under accession number of OQ931870 and OQ931871; respectively and the Phylogenetic tree including the strains was illustrated.

### Copper solutions

The metal compound CuCL_2_. 2H_2_O was formulated at concentrations of 10, 20, 50, 100 and 150 mg. L^−1^ of Cu^2+^.

### Bacterial preparation as a biosorbent

Growth of our strains supplemented with Cu^2+^ (20 mg. L^−1^). The pH of all media was adjusted to 6.8. Flasks of 100 ml were inoculated with 1 ml of overnight culture. After the growth, the method used for bacterial biomass preparation according to Hussein et al. [85].

### Effect of some factors (temperature, pH and Cu ^***2***+^ concentrations) on bioremediation and growth

Effects of different temperatures (T) (25, 30, and 35 °C) and pH values (5.0, 6.0, 7.0 and 8.0) on the bioremediation efficiency of bacteria examined using Luria–Bertani (LB) medium [86], supplemented with 20 mg. L^−1^ copper. Media with different concentrations (0, 10, 20, 50 and 100 mg. L^−1^) of Cu^+2^ were cultured with bacterial isolates to study the effect of copper concentrations on their copper removal capacity. Cultures incubated for 72 h, and the supernatant measured for amount of copper at 0, 24, 48, and 72 h. of growth. The amount of copper was determined spectrophotometrically at 325 nm, and the amount of copper removed calculated by subtracting the initial and final concentrations measured. Growth (optical density) measured at 600 nm with a UV–Vis spectrophotometer. The cell number at different time intervals was determined by determining CFU/mL for each culture.

The % removal of copper at 30 °C was defined by the estimated copper concentrations in the media containing 10 or 100 mg. L^−1^ copper. Medium containing Cu^2+^ concentration without cells used as a control for calculation of % removal.

### Determination of minimum inhibitory concentrations (MIC)

MIC of Cu-resistant bacteria were detected by dilution plat method [83]. The salts of CuCl_2_ used for preparation 1000 µg ml^−1^ stock solutions added to the medium in different concentrations (100–800 µg. ml^−1^) and then bacterial isolate inoculated into plates, and incubated for 72 h at 30 °C. The least level of copper that inhibits growth is completely deemed as MIC.

### Biosorption experiments and kinetics of sorption

The batch biosorption experiments using two bacterial isolates were achieved in 40 ml test tubes. 0.1 g (dry weight) of each type of dead cells added to 20 ml of copper solution. The tubes are then put on a shaker (150 rpm) for 24 h. Then, the samples centrifuged at 7500 rpm for 5 min. The supernatant was analyzed for residual copper concentrations using atomic absorption spectroscopy (Analytik Jena, Germany) after samples dilution.

The biosorbed amount of Cu^2+^ per gram calculated according to the following equation:

1 $${\text{q }} = \frac{{\left( {{\text{Co }}{-}{\text{ Ce}}} \right)x V}}{M}$$

where q is the biosorbed amount Cu^2+^ (mg. g^−1^ of cells), Co is the initial conc. of the Cu^2+^ (mg. ml^−1^), C_e_ is the Copper equilibrium concentration (mg. ml^−1^), V is the mixture volume (ml), and M is the cells weight (g).

To understand the adsorption process, several kinetic models have been established. The common models employed in the present study were pseudo-first order (PFO), pseudo-second order (PSO), Elovich, and intra-particle diffusion models. The generalized formulas of these models are as follows [87]**.**

2 $${\text{PFO}}: \,\,\,\,\,\,\,\,{\text{q}}_{{\text{t}}} {\text{q}}_{{\text{e}}} - {\text{exp}}^{{\left( {{\text{k}}_{{1}} {\text{t}}} \right)}}$$

3 $${\text{PSO:}}\,\,\,\,\,\,\,\,\,{\text{q}}_{t} \,\frac{{{\text{q}}_{{\text{e}}}^{{2}} {\text{k}}_{{2}} {\text{t}}}}{{{\text{1bq}}^{{2}} {\text{k}}_{{2}} {\text{t}}}}$$

Elovich equation:

4 $$qt=\frac{1}{\beta }\mathrm{ln}\left(\alpha \beta \right)+\frac{1}{\beta }\mathrm{ln}\left(t\right)$$

Intraparticle diffusion model:

5 $${\text{q}}_{{\text{t}}} = {\text{K}}_{{\text{i}}} {\text{t}}^{{0.{5}}} + {\text{ C}}_{{\text{i}}}$$

where q_e_ and qt (mg. g^−1^) are the quantities of KET or DIC adsorbed at equilibrium and at time t (min), respectively, while k_1_ (min^−1^) and k_2_ (g. mg^−1^.min^−1^) are the constants adsorption rate of the PFO and PSO models, respectively. α (mg. g^−1^.min^−1^) and β (g. mg^−1^) are the initial adsorption rate and the surface coverage, respectively, while K_i_ (mg. g^−1^.min^−1^/2) is the calculated constant of intraparticle diffusion rate.

### Modeling of adsorption isotherm

Adsorption isotherms are mathematical models that explain the actions of adsorbate species between the solid and liquid phases. To obtain more data on the adsorption mechanism, different isotherm models were applied including the Langmuir, Freundlich, Temkin, and D–R isotherms as described in the following equations [88].

Langmuir and Freundlich isotherms are used for adsorption data of Cu metals bacterial isolates.

The Langmuir mathematical formula can be stated as:

6 $${\mathrm{q}}_{\mathrm{eq}} =\frac{\mathrm{qmax }{\mathrm{bC}}_{\mathrm{eq}}}{1 + {\mathrm{bC}}_{\mathrm{eq}} }$$

The linear form of Langmuir is:

7 $${\mathrm{C}}_{\mathrm{eq}}/{\mathrm{q}}_{\mathrm{eq}}=\frac{1}{\mathrm{qmax b}}+\frac{{\mathrm{C}}_{\mathrm{eq}}}{\mathrm{qmax}}$$

where, q_max_ is the maximum metal adsorption capacity per unit weight biomass, and b (L. mg^−1^) is Langmuir constant.

By Freundlich isotherm, the correlation between the dissolved and adsorbed copper concentration was calculated. The Freundlich isotherm linear form is expressed by:

8 $$\mathrm{log q}=\mathrm{log K}+\frac{1}{\mathrm{ n log C}}$$

where q is the adsorbed amount of Cu^2+^ per gram sorbent (mg. g^−1^), C is the adsorbate equilibrium concentration (mg. ml^−1^), and K and n are constants of Freundlich (adsorption capacity and intensity; respectively). Parameters of Freundlich be able to obtain by plotting log Q vs. log C, with 1/n being the slope and log K being the intercept of the line.

#### Temkin isotherm

9 $$q_{e} = BT\ln AT + {\text{BT lnC}}_{{\text{e}}}$$

where BT ¼ RT = bT T is the absolute temperature (298.15 K), bT is related to the heat of sorption (J mol^−1^), AT is the equilibrium constant binding relating to the maximum energy binding, and BT is the Temkin isotherm constant (L. mg^−1^).

#### D-R isotherm

10 $$\ln \left( {q_{e} } \right) = \ln \left( {Q_{s} } \right) - \beta {\mathcal{E}}^{2}$$

Where

11 $$\varepsilon \, = \,\raise.5ex\hbox{$\scriptstyle 1$}\kern-.1em/ \kern-.15em\lower.25ex\hbox{$\scriptstyle 4$} {\text{ RTln }}\left( {{1}\, + \,{1 }/{\text{C}}_{{\text{e}}} } \right)$$

Ce is the equilibrium concentration of the pollutant (mg. L^−1^), q_e_ is the maximum adsorbed amount (mg. g^−1^). R is the universal gas constant (8.314 J mol^−1^.K^−1^), T is the absolute temperature (298.15 K).

### Statistical analyses

Statistical analysis of the data was conducted using ANOVA one-way test (analysis of variance) by SPSS program version 21, and Duncan values were determined at 0.05 levels.

All the experiments were performed in triplicate. The solver function in the Microsoft Excel software was used in the nonlinear regression analysis for fitting the experimental data to the different kinetic and isotherm equations.

## Conclusions

The current study mainly focuses on biosorption of copper by *Bacillus pumilus* OQ931870 and *Bacillus subtilis* OQ931871 which isolated from Wadi Nakheil, Red Sea, Egypt. Copper biosorption equilibrium data have been fitted well to the Langmuir and Freundlich models for the two strains. Pseudo-first and second order kinetic models provided a better explanation of the adsorption kinetics. The Temkin isotherm and the Elovich kinetic model indicated that the bacterial surface is heterogeneous. The D-R isotherm suggested that the adsorption process was more oriented toward physical adsorption, the intra-particle diffusion model proposed a boundary layer effect. Furthermore, the important and effective parameters of Cu ^2+^ biosorption are the pH, temperature, time and copper concentrations. The results of the current investigation show that our isolates may be used to successfully copper remove from the aquatic ecosystem at low cost and with no environmental impact.

## Data Availability

The data will be provided under request.
